# Response to: Comment on “Failure of the Pipeline Embolization Device in Posterior Communicating Artery Aneurysms Associated with a Fetal Posterior Cerebral Artery”

**DOI:** 10.1155/2017/9895414

**Published:** 2017-05-30

**Authors:** Mario Zanaty, Nohra Chalouhi, Robert M. Starke, Pascal Jabbour, Katherine O. Ryken, Ketan R. Bulsara, David Hasan

**Affiliations:** ^1^Department of Neurosurgery, University of Iowa Hospital and Clinics, 200 Hawkins Drive, Iowa City, IA 52242, USA; ^2^Department of Neurological Surgery, Thomas Jefferson University and Jefferson Hospital for Neuroscience, 3rd Floor, 901 Walnut Street, Philadelphia, PA 19107, USA; ^3^Department of Neurosurgery, University of Virginia, Charlottesville, VA 22908, USA; ^4^Department of Neurosurgery, Yale University, New Haven, CT 06519, USA

We would like to thank Dr. Srinivasan and Dr. Kan for their remarks [1] on our paper [2]. We think that these publications were developed simultaneously. In addition, we think that more series would be reported in the future regarding the failure of the pipeline in the treatment of posterior communicating artery aneurysms associated with a fetal posterior cerebral artery. We also congratulate Srinivasan et al. [3] and Kan et al. [4] on their work on this topic and we agree that the most likely explanation of the pipeline failure is the high flow demand in this type of circulation. This can also explain why parent artery occlusion can lead to cerebellar infarction in this subset of patients [5]. We are also working on reporting other aneurysm geometrical factors that contribute to failure of flow-diversion; however this work is still in its early stages.
